# CompGO: an R package for comparing and visualizing Gene Ontology enrichment differences between DNA binding experiments

**DOI:** 10.1186/s12859-015-0701-2

**Published:** 2015-09-02

**Authors:** Ashley J. Waardenberg, Bassett Maya, Romaric Bouveret, Richard P. Harvey

**Affiliations:** 1 Victor Chang Cardiac Research Institute, Darlinghurst, NSW 2010 Australia; 2 Present Address: Children’s Medical Research Institute, Westmead, NSW 2145 Australia; 3 St. Vincent’s Clinical School, University of New South Wales, Kensington, 2052 Australia; 4 School of Biotechnology and Biomolecular Sciences, University of New South Wales Faculty of Science, New South Wales, 2052 Australia; 5 Stem Cells Australia, Melbourne Brain Centre, University of Melbourne, Victoria, 3010 Australia

**Keywords:** Gene Ontology, Serum Response Factor, Jaccard Coefficient, Enrich Gene Ontology, Hard Thresholding

## Abstract

**Background:**

Gene ontology (GO) enrichment is commonly used for inferring biological meaning from systems biology experiments. However, determining differential GO and pathway enrichment between DNA-binding experiments or using the GO structure to classify experiments has received little attention.

**Results:**

Herein, we present a bioinformatics tool, CompGO, for identifying Differentially Enriched Gene Ontologies, called DiEGOs, and pathways, through the use of a z-score derivation of log odds ratios, and visualizing these differences at GO and pathway level. Through public experimental data focused on the cardiac transcription factor NKX2-5, we illustrate the problems associated with comparing GO enrichments between experiments using a simple overlap approach.

**Conclusions:**

We have developed an R/Bioconductor package, CompGO, which implements a new statistic normally used in epidemiological studies for performing comparative GO analyses and visualizing comparisons from .BED data containing genomic coordinates as well as gene lists as inputs. We justify the statistic through inclusion of experimental data and compare to the commonly used overlap method. CompGO is freely available as a R/Bioconductor package enabling easy integration into existing pipelines and is available at: http://www.bioconductor.org/packages/release/bioc/html/CompGO.html packages/release/bioc/html/CompGO.html

## Background

Gaining biological insight from high-throughput data underpins systems biology. However, determining biological “function” or indeed “relevance” from lists of genes or DNA regions (loci) remains problematic. Ashburner et al. proposed a structured Gene Ontology (GO) approach for grouping genes into conceptual “ontologies” based on their annotated or predicted biological functions [1]. GOs are organized into a hierarchical network where broad functionality sits at the top (e.g. cell) and fine functionality at the bottom (e.g. calcium ion binding). Individual genes can have multiple GOs. The accumulation of gene annotations and subsequent classification of thousands of ontologies has seen the development of a number of tools using a range of statistical approaches to identify “semantic” patterns, or GO enrichment, within a given list of genes [2]. GO enrichment is typically determined using a hypergeometric test (or modified version) or similar over-representation test based on gene sets alone or, for example, signatures derived from the correlation of gene expression profiles [3–5].

However, few methods have been developed to determine how similar or different experiments are using a GO approach; most are focused on different visualization methods and are not adaptable to existing pipelines, requiring users to reformat and manually input data into third party web services. For instance, WebGestalt [6] and GOEAST [7] are webservers that visualize multiple gene list inputs by overlaying their individual statistics onto a GO directed acyclic graph. Enrichment maps visualize GO enrichment from multiple gene lists as a network; edges derived from the Jaccard coefficient (JC) of GO gene set overlap [8]. However, enrichment maps are difficult to resolve when more than two experiments are compared and do not indicate overall differences between experiments. Comparative GO [9], a web based GO tool, via the Kolmogorov-Smirnov statistic, compares observed GOs to an expected GO distribution, however is limited to bacterial gene lists and visualization of pairwise comparisons.

Motivated by our interest in DNA binding experiments (e.g. ChIP-seq or DamID) and their similarities/differences, we developed a tool that would enable rapid comparison of multiple experiments unconstrained by input type (gene list or loci) or species, and taking advantage of existing unsupervised clustering and dimensionality reduction methods (e.g. hierarchical clustering and principle component analysis), implemented in R for classification of experiments based on GO. We present an open-source implementation of a comparative GO approach, CompGO, which is readily adaptable to existing analysis pipelines for performing these functions and implement a log odds ratio [10, 11] normally applied to epidemiological studies for comparing GO enrichment directly. We justify the use of this statistic for direct comparisons by assessing experimental data recently published [12].

## Implementation

### GO enrichment

**Fig. 1:**
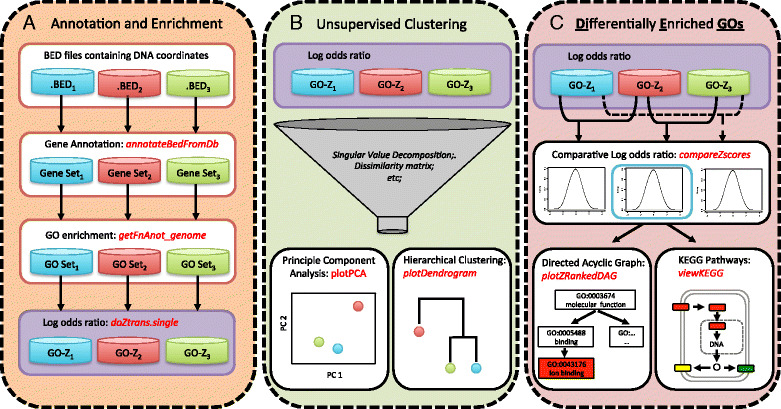
Overview of the CompGO pipeline and implemented functions. **a** The “annotateBedFromDb” function annotates DNA coordinates from BED files against transcript coordinates from a reference genome, “getFnAnot_genome” queries gene lists using the RDAVIDWebService and returns statistics and counts of each GO term and “doZtrans.single” calculates the log odds ratio of GO term enrichment. Note: users can supply their own background genome regions; by default the whole genome is used. **b** Given log odds ratios, multiple experiments can be reduced into a single matrix for Principle Component Analysis or Hierarchical Clustering, via “plotPCA” and “plotDendrogram” respectively. **c** Differentially Enriched GOs (DiEGOs) between pairs of experiments are calculated via the differential log odds ratio and top DiEGOs can be visualized via Directed Acyclic Graphs, “plotZRankedDAG”, and top differentially enriched pathways via “viewKEGG”. CompGO functions are colored red

We developed an R package, CompGO, to assess similarities and differences between experiments using a log odds ratio scoring (z-score) [10, 11] of GO enrichment (Eqs. 1–4); the pipeline is outlined in Fig. 1. CompGO is compliant to R/Bioconductor [13] standards (available in Bioconductor version 2.14 onwards) and therefore takes advantage of prebuilt statistical and visualization functions already included in R [14]. CompGO enables users to input data of either annotated gene symbols/identifiers or BED file format. CompGO utilizes existing packages in Bioconductor, rtracklayer, to annotate loci using transcript coordinates derived from UCSC genome databases [15], RDAVIDWebService [16] to interrogate the DAVID GO database and KEGG.db to visualize enrichment of annotated pathways [17]. We use DAVID (The Database for Annotation, Visualization and Integrated Discovery) [4] as a GO reference dataset, but the principles and method could be applied to any GO database.

### Differential GO enrichment

GO count data was derived from the 2x2 contingency table for each GO term returned by RDAVIDWebService. In addition to the statistics returned by DAVID, we implement a log odds-ratio, δ, [10, 11] scoring for determination of GO enrichment (Eq. 1). Extension to a comparative log odds-ratio (Eq. 3) enables differential GO enrichment for each GO term to be calculated by direct comparison of 2x2 contingency tables derived from different experiments, also enabling comparison of experiments with different background distributions or coverage. Here z_i_ is the z-score for the i-th GO term:

1 $$ {z}_i=\frac{ \log \left({\delta}_i\right)}{SE\left({\delta}_i\right)} $$

given a Standard Error, SE (δ_i_), for each term, i, where n_1_ to n_4_ are the four components (observed counts, total genes, background counts, background genes tested) of the i-th 2x2 contingency table.

2 $$ SE\left({\delta}_i\right)=\sqrt{\frac{1}{n_1}+\frac{1}{n_2}+\frac{1}{n_3}+\frac{1}{n_4}} $$

p-values are not derived from log odds ratios, but 95 % confidence intervals could be assigned to enrichment scores as z_i_ ± 1.96SE (δ_i_). The greater the absolute z_i_, the greater the odds a term was enriched than by chance alone.

When computing differential enrichment between two sets of GO terms, we employ a comparative log odds ratio, z_k_, derived from the difference of two non-zero log odds ratios, log (δ_i_) and log (δ_j_), for the k-th term:

3 $$ {z}_k=\frac{ \log \left({\delta}_i\right)- \log \left({\delta}_j\right)}{SE\left({\delta}_{ij}\right)} $$

given the total Standard Error, SE (δ_ik_), for each term, k, where SE_i_ and SE_j_ are derived as per Eq. 2:

4 $$ SE\left({\delta}_{ij}\right)=\sqrt{S{E}_i^2+S{E}_j^2} $$

Scoring of Differentially Enriched Gene Ontologies (DiEGOs) can then be inferred from their z-scores. The greater the absolute z_k_, the greater the odds a term was differentially enriched than by chance alone. p-value’s can be inferred using R assuming normal approximations and multiple methods are available for correcting for multiple hypotheses.

### Overlap of genes between GOs

To assess gene overlap within a GO category (enriched in two gene lists), we utilize the Jaccard coefficient (JC) [18] of any two gene sets (A, B) from two comparisons contributing to term k. We include this statistic as similar levels of GO enrichment can be achieved between experiments even though the genes contributing to a GO can be distinct. The JC is the ratio of the intersection and the union of these sets:

5 $$ JC=\frac{A\cap B}{A\cap B} $$

### Example of CompGO Code

**Fig. 2:**
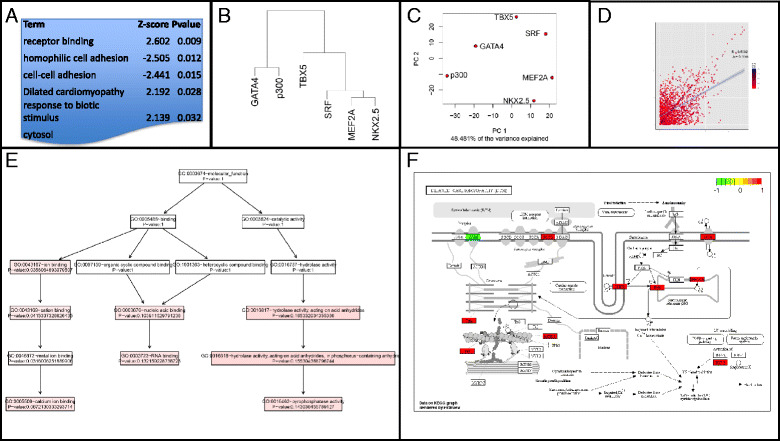
Example functionality of CompGO using published ChiP-seq data. 1000 BED coordinates were selected at random and form part of the example dataset packages with CompGO. **a** Differentially enriched GO and pathway terms. **b** Hierarchical clustering 1. **c** Principle Component Analysis. **d** Direct comparison of z-scores with Jaccard Coefficient overlaid (Eq. 5) onto terms. **e** Directed Acyclic Graph. **f** KEGG Pathway colored by which experiment the Gene was mapped to. **a**, **b**, **e** and **f** utilise Eq. 3 in their rankings. **d** utilises Eq. 1


For illustration purposes, an example dataset was produced by randomly selecting 1000 BED coordinates from published ChiP-seq data of different transcription factors (TFs) and their co-factors profiled in cultured HL-1 cardiomyocytes: NKX2-5, MEF2A, GATA4, p300, SRF and TBX5 [19]. This example data is included with the CompGO package and example code for running core CompGO functions is provided below (Fig. 2a-f) . For more example code and updated functionality, see the CompGO Reference Manual and accompanying Vignette on the Bioconductor website.

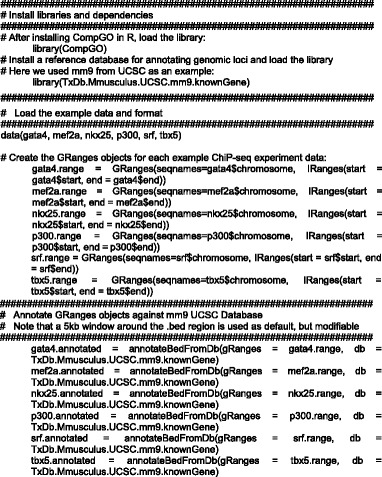


## Results and discussion

To determine the utility of the methods proposed in CompGO we downloaded DNA targeted regions (peaks) for a number of wild-type (WT) and mutated cardiac TFs identified by Bouveret et al. [12] using the DamID method, and compare the outcomes using a simple overlap approach. Bouveret et al. surveyed DNA binding regions for the WT NKX2-5 cardiac transcription factor twice (independent experiments with 3–4 replicates each performed 2 years apart; data sets hereafter named NKX2-5_1_ and NKX2-5_2_) and in addition surveyed three NKX2-5 mutants - NKX2-5Y191C is a congenital heart disease-causing mutation [20, 21], while NKX2-5ΔHD and NKX2-5YRD^Y-A^ are synthetic mutations with a disrupted homeodomain (involved in both DNA-binding and cofactor interactions) and Tyrosine-Rich Domain (YRD; cofactor interactions), respectively. DNA binding regions of the muscle-enriched TF serum response factor (SRF) and the ubiquitously-expressed ETS-domain TFs ELK1 and ELK4, were also considered [12] .

**Fig. 3:**
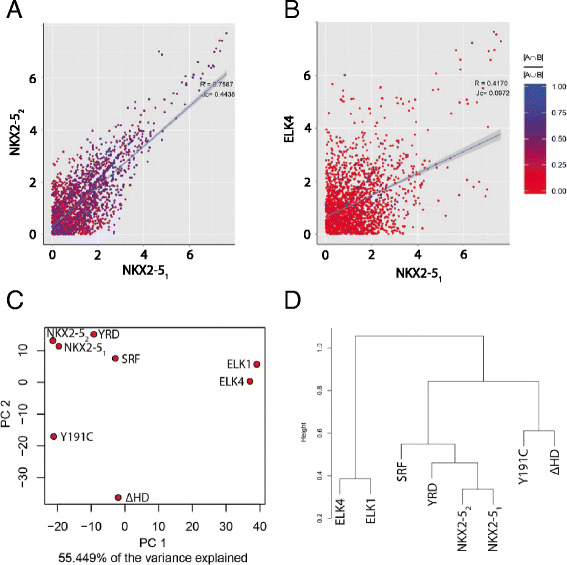
Application of CompGO to experimental data. Direct comparison of z-scores with Jaccard Coefficient overlaid onto terms for **a** NKX2-5_1_ vs. NKX2-5_2_ and **b** NKX2-5_1_ vs. ELK4. **c** Principle Component Analysis. **d** Hierarchical clustering

All results reported were using CompGO default settings. Peak coordinates were annotated and GO term counts obtained as per the process outlined in Fig. 1a. Direct comparison of z-score-transformed GO enrichments (Eq. 1) illustrated that repeated NKX2-5 experiments were highly correlated (*R* = 0.76) and had a high average JC (0.44) (Fig. 3a) of individual GO terms in contrast to ELK4 (*R* = 0.42; JC = 0.10; Fig. 3b), ELK1 (*R* = 0.39; JC = 0.10), SRF (*R* = 0.62; JC = 0.18) or NKX2-5 mutations (*R* = 0.47-0.67; JC = 0.15-0.40) (graphical representations not shown). Unsupervised principle component analysis and hierarchical clustering placed NKX2-5_1_ and NKX2-5_2_ next to each other and close to SRF and the NKX2-5YRD^Y-A^ mutation, while ELK1 and ELK4, and the other NKX2-5 mutations, were located at greater distance (Fig. 3c and d). The related ELK TFs were also placed next to each other. We then computed DiEGOs as per Eq. 3 for each comparison. Using a p-value threshold of 0.05 we did not identify any DiEGOs for the two repeated NKX2-5 experiments, but identified 43/44, 31/37, 18/21, 1/11, 15/10 and 0/0 DiEGOs when comparing NKX2-5_1_/NKX2-5_2_ to ELK4, ELK1, NKX2-5ΔHD, SRF, NKX2-5Y191C and NKX2-5YRD^Y-A^, respectively.

These results suggest that ELK TFs regulate distinct although overlapping sets of biological processes compared to NKX2-5. Furthermore, while SRF and the mutation NKX2-5YRD^Y-A^ largely target genes with similar GO terms as WT NKX2-5, the mutations NKX2-5ΔHD and NKX2-5Y191C, predicted to be the more severe mutations among those studied here, targeted sets of genes representing distinct biological processes [12]. Notably the average JC, a metric representing overall concordance of genes belonging to the same GO term, varied, indicating that distinct sets of target genes could belong to the same GO term. Of the DiEGOs from the NKX2-5_1_ versus ELK4 comparison, those unique to ELK4 included metabolic and generic GO terms such as GO:0006396 ~ RNA processing (z-scores: 0.13 vs. 5.41; p-value: 0.001) and GO:0034470 ~ ncRNA processing (z-scores: -0.09 vs. 3.60; p-value: 0.028), whereas those for NKX2-5_1_ included muscle related terms such as GO:0043292 ~ contractile fiber (z-scores: 6.50 vs. 1.70; p-value: 0.035) and GO:0048514 ~ blood vessel morphogenesis (z-scores: 4.00 vs. 0.26; p-value: 0.043). This is consistent with the known roles for NKX2-5 in muscle and vasculature development and the ubiquitous expression of ELK TFs [22].

**Fig. 4:**
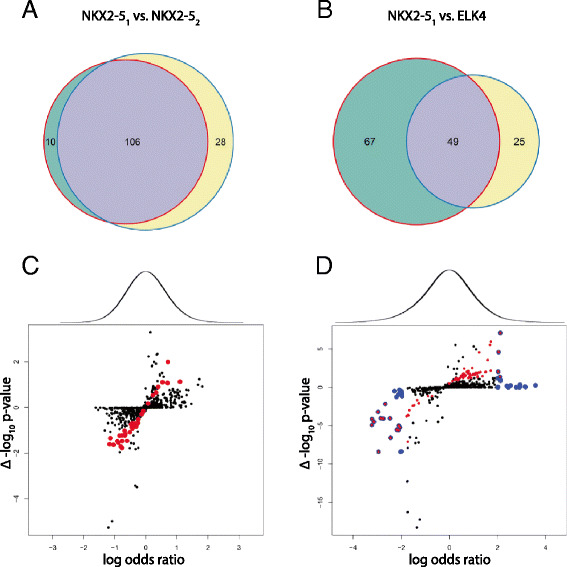
Comparison of CompGO to the overlap method. Differentially enriched GO terms using the overlap method (*p* ≤ 0.05) for **a** NKX2-5_1_ vs. NKX2-5_2_ and **b** NKX2-5_1_ vs. ELK4. **c** Log odds ratio of NKX2-5_1_ vs. NKX2-5_2_ versus differential p-values returned from DAVID, scored as the difference between –log10 transformed vales. **d** Log odds ratio of NKX2-5_1_ vs. ELK4 versus differential p-values returned from DAVID, scored as the difference between –log10 transformed vales. Red dots indicate GO terms determined significant and specific using the overlap method. Blue dots are GO terms returned as significant from CompGO. Blue dots with red centres are GO terms returned as significant by both methods. Black dots are non-significant terms using both approaches. For (**b**) and (**c**) the density distribution of log odds ratios returned by CompGO is on the top of each panel

We then compared results of 1) NKX2-5_1_ versus NKX2-5_2_; and 2) NKX2-5_1_ versus ELK4 using a simple overlap method of thresholding each GO term (*p* < 0.05) using the statistic returned by DAVID (Benjamini & Hoschberg adjusted). This reported 38 GO terms as being specifically enriched in either group for NKX2-5_1_ versus NKX2-5_2_ (Fig. 4a) and 92 for NKX2-5_1_ versus ELK4 (Fig. 4b). However, upon closer inspection many of the differences could be attributed to “hard thresholding”. That is, many GO terms in the comparison experiment had a significance value just beyond the 0.05 threshold imposed, falsely making it appear to be differentially enriched due to the selection of the significance threshold. In addition, many of the GO terms only changed their group membership by a few genes. For example, “GO:0003824 ~ catalytic activity” would have been reported as differentially enriched using this overlap approach, having a p-value of 0.066 in one experiment and 0.011 in the other, whilst only changing counts by less than 1 %, from 420 to 417. However, this effect was more pronounced in the lower count range. For example, “GO:0044448 ~ cell cortex part” reported a p-value of 0.420 in one experiment and 0.025 in the other, whilst only changing counts from 10 to 14. Both of these examples were reported as non-significant when directly compared using the log odd ratios proposed in Eq. 3 with p-values of 0.763 and 0.399, respectively. This suggests that differences observed using the overlap method are likely to be false-positives as a consequence of specificity issues (i.e. proportion of correctly classified negative results).

To better illustrate the differences, we compared the overlap method to the log odds ratio method by directly computing the differential of p-values (scored as the difference between –log_10_ transformed p-values or simply ‘Δ –log_10_ p-value’) returned by DAVID to the log odds ratio returned from direct comparison using CompGO for NKX2-5_1_ versus NKX2-5_2_ (Fig. 4c) and NKX2-5_1_ versus ELK4 (Fig. 4d). For NKX2-5_1_ versus NKX2-5_2_, this illustrated that GO terms reported by the overlap method did not approximate to the tails of the distribution where differences would be expected to occur if compared directly as per the log odds ratio in Eq. 3. When comparing NKX2-5_1_ to ELK4 some concordance was observed, but there was still a large number of differentially enriched GO terms identified using CompGO that were 1) not detected using the overlap method; and 2) not approximating to the tails of the log-odds distribution - likely to be false positives (Fig. 4d). In addition to hard thresholding, DieGOs identified by CompGO and not detected using the overlap method arose as a result of “under-representation”. This is because the log odds ratio (Eq. 3) considers both tails of the distribution, in contrast to the single-tailed modified Fishers exact test implemented in DAVID which only considers over-representation. For example, DAVID returned p-values of 0.54 and 1.00 for GO:0006811 ~ ion transport indicating that this GO term was not significantly over-represented in either set, however CompGO returned a p-value of 0.0003 which reflected an under-representation of this term for ELK4 targets (z-scores: 1.57 vs. -3.23). Therefore, the approach of hard thresholding of individual GO statistical results from each comparison and performing overlaps introduces many false positives as well as missing potential differences. This illustrates how CompGO overcomes the issue of hard thresholding implicit in the overlap method by directly computing differential enrichment via a log odds ratio, thereby reducing the number of false positive results.

## Conclusions

CompGO enables rapid identification, comparison and visualization of differentially enriched GO terms calculated from multiple lists of genetic loci. Through experimental data we illustrate the problems associated with comparing GO enrichment between experiments using a simple overlap method in contrast to the proposed log odds ratio. CompGO provides methods to address the questions of “how significant are GO enrichment differences?” and “how similar are multiple experiments based on GO enrichments”. Input data can be .BED files or gene identifiers. CompGO is applicable to any species where a reference genome assembly is available. As CompGO is implemented in R, it is accessible to a broad range of users and can readily be incorporated into existing pipelines. CompGO is an easy and fast comparative package for GO enrichments from experimentally identified DNA regions or genes.

## Availability

**Project name:** CompGO

**Project home page:** http://www.bioconductor.org/packages/release/bioc/html/CompGO.html

**Operating system(s):** Platform independent

**Programming language:** R

**Other requirements:** BioC 2.14 (R-3.1)

**License:** GPL-2

## Abbreviations

GO: Gene Ontology
DiEGOs: Differentially Enriched Gene Ontologies
JC: Jaccard coefficient
DAVID: The Database for Annotation, Visualization and Integrated Discovery
HD: NKX2-5 homeodomain
YRD: NKX2-5 tyrosine-rich domain
TFs: Transcription factors
