# Erratum to: ‘CompGO: an R package for comparing and visualizing Gene Ontology enrichment differences between DNA binding experiments’

**DOI:** 10.1186/s12859-016-1048-z

**Published:** 2016-04-25

**Authors:** Ashley J. Waardenberg, Samuel D. Bassett, Romaric Bouveret, Richard P. Harvey

**Affiliations:** Victor Chang Cardiac Research Institute, Darlinghurst, NSW 2010 Australia; Present Address: Children’s Medical Research Institute, Westmead, NSW 2145 Australia; St. Vincent’s Clinical School, University of New South Wales, Kensington, 2052 Australia; School of Biotechnology and Biomolecular Sciences, University of New South Wales Faculty of Science, New South Wales, 2052 Australia; Stem Cells Australia, Melbourne Brain Centre, University of Melbourne, Victoria, 3010 Australia

Unfortunately, the original version of this article [1] contained an error. The author’s family name was spelt incorrectly as ‘Basset’. We have included it here correctly as ‘Bassett’ for reference. The original article will be corrected to reflect this.
